# ASO Author Reflections: Augmenting the Future Liver Remnant Prior to Major Hepatectomy: A Review of Options on the Menu

**DOI:** 10.1245/s10434-025-17679-x

**Published:** 2025-06-13

**Authors:** Paul Wong, Laleh G. Melstrom

**Affiliations:** Division of Surgical Oncology, Department of Surgery, City of Hope Medical Center, Duarte, CA USA

## Past

Post-hepatectomy liver failure remains a significant clinical challenge and a major contributor to morbidity and mortality after major liver resection.^1^ For both primary and secondary liver malignancies, this critical issue has been addressed by optimizing the future liver remnant (FLR). Through the years, various strategies have been introduced to induce hypertrophy of the FLR before major hepatectomy, including portal vein embolization (PVE), portal vein ligation (PVL), liver venous deprivation (LVD), association of liver partition and portal vein ligation for staged hepatectomy (ALPPS), and radioembolization (e.g., Y-90). However, there remains variability in techniques, heterogeneity in retrospective studies, and a paucity of controlled comparisons, which have made it difficult to draw definitive conclusions about the most effective approach.

## Present

This review article by Wong et al.^2^ synthesizes the current evidence surrounding these FLR augmentation techniques, outlining their respective benefits and limitations with regard to hypertrophy efficacy, perioperative risk, and oncologic outcomes. It critically examines existing comparative studies and ongoing randomized controlled trials, emphasizing the need for a nuanced understanding of these strategies to inform clinical decision-making. By highlighting gaps in head-to-head comparisons and the importance of tailoring approaches to patient-specific factors, this review contextualizes current practices and serves as a valuable resource for clinicians managing complex liver surgery cases.

## Future

Importantly, the discussion of FLR augmentation is awaiting results of ongoing randomized controlled trials, especially the DRAGON 2, HYPER-LIV01, DRAGON-PLC trials, which will investigate the comparative efficacy of LVD and PVE.^3–5^ The completion of these studies and subsequent long-term follow-up evaluation will provide Level 1 evidence to inform the selection of the best strategy regarding capacity for sufficient hypertrophy for resection, perioperative morbidity/mortality, and oncologic outcomes. However, further research should aim to develop personalized algorithms that incorporate patient comorbidities, tumor biology, and liver function to adopt the most appropriate hypertrophy strategy. Integrating these findings into clinical practice may help standardize decision-making and improve both surgical candidacy and survival for these subsets of patients. The goal is to achieve the most optimal outcome for the patient with the least morbidity.
